# Decomposition of Near-Infrared Spectroscopy Signals Using Oblique Subspace Projections: Applications in Brain Hemodynamic Monitoring

**DOI:** 10.3389/fphys.2016.00515

**Published:** 2016-11-08

**Authors:** Alexander Caicedo, Carolina Varon, Borbala Hunyadi, Maria Papademetriou, Ilias Tachtsidis, Sabine Van Huffel

**Affiliations:** ^1^Department of Electrical Engineering ESAT, STADIUS Center for Dynamical Systems, Signal Processing, and Data Analytics, KU LeuvenLeuven, Belgium; ^2^(iMinds Medical) Department Medical Information TechnologiesLeuven, Belgium; ^3^Biomedical Optics Research Laboratory, Department of Medical Physics and Bioengineering, University College LondonLondon, England

**Keywords:** oblique subspace projections, Tikhonov regularization, biomedical signal processing, NIRS, brain hemodynamics

## Abstract

Clinical data is comprised by a large number of synchronously collected biomedical signals that are measured at different locations. Deciphering the interrelationships of these signals can yield important information about their dependence providing some useful clinical diagnostic data. For instance, by computing the coupling between Near-Infrared Spectroscopy signals (NIRS) and systemic variables the status of the hemodynamic regulation mechanisms can be assessed. In this paper we introduce an algorithm for the decomposition of NIRS signals into additive components. The algorithm, SIgnal DEcomposition base on Obliques Subspace Projections (SIDE-ObSP), assumes that the measured NIRS signal is a linear combination of the systemic measurements, following the linear regression model **y** = **Ax** + **ϵ**. SIDE-ObSP decomposes the output such that, each component in the decomposition represents the sole linear influence of one corresponding regressor variable. This decomposition scheme aims at providing a better understanding of the relation between NIRS and systemic variables, and to provide a framework for the clinical interpretation of regression algorithms, thereby, facilitating their introduction into clinical practice. SIDE-ObSP combines oblique subspace projections (ObSP) with the structure of a mean average system in order to define adequate signal subspaces. To guarantee smoothness in the estimated regression parameters, as observed in normal physiological processes, we impose a Tikhonov regularization using a matrix differential operator. We evaluate the performance of SIDE-ObSP by using a synthetic dataset, and present two case studies in the field of cerebral hemodynamics monitoring using NIRS. In addition, we compare the performance of this method with other system identification techniques. In the first case study data from 20 neonates during the first 3 days of life was used, here SIDE-ObSP decoupled the influence of changes in arterial oxygen saturation from the NIRS measurements, facilitating the use of NIRS as a surrogate measure for cerebral blood flow (CBF). The second case study used data from a 3-years old infant under Extra Corporeal Membrane Oxygenation (ECMO), here SIDE-ObSP decomposed cerebral/peripheral tissue oxygenation, as a sum of the partial contributions from different systemic variables, facilitating the comparison between the effects of each systemic variable on the cerebral/peripheral hemodynamics.

## 1. Introduction

Signal decomposition methods aim at representing a signal as a combination of components that fulfill some specific criteria. For instance, a wavelet transform decomposes a signal into a set of time series that are localized both in frequency and time. Generally, these decomposition schemes define a subspace using an orthonormal basis onto which the signal to be decomposed is projected. In this way the signal is represented in the transformed subspace as a linear combination of the given basis vectors. These decomposition schemes can be seen as a linear regression problem of the form **y** = **Ax** + **ϵ**, where **y** represents the signal of interest, **A** is a matrix containing the basis for the subspace defined by the transformation, **x** is a vector containing the decomposition coefficients that represent the signal in the transformed domain, and **ϵ** represents the error term.

Signal decomposition methods can also be linked to the field of system identification, where the output of a system is computed as a linear combination of the partial contributions of the input variables (Ljung, 1999). An identification problem can be formulated as a regression problem of the same form mentioned above, **y** = **Ax** + **ϵ**, which is a matrix representation of a convolution operation, with **x** being the impulse response of the system. However, in this case, the matrix **A** is constructed differently, and does not contain an orthonormal basis representing the transformed subspace. Instead, this matrix is created from input-output observations of the system using a specific model form to fit the output variable. Models such as ARX (autoregressive with exogenous input), ARMAX (autoregressive moving average with exogenous input), ARIMA (autoregressive integrative moving average), among others have been extensively studied in the literature (Ljung, 1999). These models can be used for simulation, as well as prediction of the system output **y**, e.g., in the prediction of the brain hemodynamic response to an stimulus (Aqil et al., 2012). Others examples of the use of this models in the field of near infrared spectroscopy can be found in Hong and Naseer (2016), Kamran and Hong (2013), Pillonetto (2016), Kamran and Hong (2014), Naseer and Hong (2015). However, the main focus of these models is not to compute the individual contribution of each input variable on the output, but to produce a good estimation of the behavior of the system as a whole, which leads to a lack of interpretability of the models in terms of the underlying physiological processes.

Since real-life problems are likely to be characterized by correlated inputs, the system subspace will consist of a set of non-orthogonal subspaces, challenging the identification of individual contributions, as well as obscuring its clinical interpretation. For instance, when using least squares to identify the contribution of a single subsystem on the output, the noise is assumed to be orthogonal to the system subspace, which due to the presence of correlated inputs is clearly not the case, hence producing erroneous estimates. This problem can be mitigated by the use of an appropriate projector that allows a more effective separation of the different subsystems' dynamics, such as an oblique subspace projector.

Oblique subspace projectors (ObSP) are projection matrices that use a reference subspace trajectory to project a signal onto a desired subspace. When the reference subspace is orthogonal to the desired subspace ObSP reduces to an orthogonal projector. ObSP possesses properties that can be exploited to produce an appropriate decomposition of biomedical signals, in terms of the linear contributions of the different input variables. This allows to provide physical interpretation to the system, in the framework of identification models.

Specifically, this kind of decomposition framework can be used to decipher the relation between different biomedical signals, helping to understand their relations, as well as facilitating their clinical interpretation. In particular in the current clinical practice where patients measurements comprise a large number of concomitant measurements of different biomedical signals, ObSP can aid in facilitating a more accurate interpretation of physiological or patho-physiological processes, since the influence of each input variable on the output can be analyzed separately without interference from confounding factors, this is a critical factor for the clinical interpretability of mathematical models (Slinker and Glantz, 1985). For instance, for the assessment of cerebral autoregulation in premature infants surrogate measurements of cerebral blood flow (CBF), obtained using Near-infrared Spectroscopy (NIRS), can be used. But only when there are not strong variations in SaO_2_ (Soul et al., 2007; Wong et al., 2008). However, NIRS measurements are highly coupled to changes in arterial oxygen saturation (SaO_2_), which introduces information that is not directly linked to changes in CBF. This hinders the use of NIRS as a technology for the bed-side assessment of cerebral autoregulation. In order to correctly assess the status of the cerebral autoregulation mechanism, using NIRS, changes in SaO_2_ should be decoupled from the NIRS measurements, prior to further processing. In such an example the use of a decomposition framework, as the one presented in this paper, becomes relevant since it can be used as a preprocessing step to decouple the influences of changes in SaO_2_ from the NIRS signals.

Applications of ObSP are scarce in signal processing. Among the applications we can find in the literature we list the work from Behrens and Scharf (1994), who presents the use of ObSP for interpolation, decoding, and elimination of symbol interferences in a communication channel. Tu et al. (1999), used ObSP for hyperspectral image classification. They applied ObSP to quantify the mixture of spectral signatures, from different materials, contained in a specific pixel of an hyperspectral image. Van Overschee and De Moor (1994) proposed the used of subspace system identification, which intrinsically uses ObSP in order to estimate the state space of the system. In the biomedical signal processing field, in some previous work, we have proposed the use of ObSP in combination with wavelet decomposition for cerebral hemodynamics monitoring (Caicedo et al., 2012). There, we aimed at decomposing cerebral hemodynamic signals, measured by means of NIRS, as a sum of the partial linear contributions of different systemic variables such as, mean arterial blood pressure (MABP), SaO_2_, heart rate (HR), end tidal CO_2_, among others. We also proposed the use of ObSP as a preprocessing method for NIRS measurements (Caicedo et al., 2013a), and for the extraction of features in a sleep apnea detection algorithm (Varon et al., 2015). In this paper we introduce the theoretical framework for a decomposition algorithm based on ObSP which can be applied in the field of cerebral hemodynamic monitoring. In addition we impose smoothness in the regression parameters by the use of Tikhonov regularization using a matrix differential operator, in contrast with the wavelet decomposition framework of our previous work, resulting in a more formal and flexible problem definition, a more robust solution, as well as a more clear decomposition framework. We test the performance of ObSP with a synthetic example as well as with 2 application examples in the biomedical field, specifically related to the monitoring of brain hemodynamics regulation. In the first application example we decouple the changes in SaO_2_ from the NIRS recordings, the SaO_2_ is measured using a pulse oxymeter. In the second application example, we find the partial linear contributions of each input variable on the changes of cerebral and peripheral tissue oxygenation. In this example we use as input variables concomitant measurements of MABP, EtCO_2_, HR, SaO_2_, and ECMO flow.

The rest of the paper is organized as follows. In Section 2 we briefly introduce ObSP. In Section 2.1 we present the geometrical interpretation for ObSP, and we propose to solve the ObSP problem by solving an alternative least squares problem. The new problem definition allows to smoothly introduce Tikhonov regularization. In Section 3 we present the proposed general algorithm for signal decomposition using ObSP. In Section 4 we show the results from the synthetic and applications examples. Finally, in Sections 5, 6 we discuss our main findings and present the concluding remarks.

Along the manuscript we will represent scalars by lowercase variables such as *x*. Vectors will be represented by bold lowercase variables such as **x**. Matrices will be represented by bold uppercase variables such as **A**. Generic vector subspaces will be represented by blackboard bold letters such as ℝ, and vector subspaces generated by a given matrix will be represented using calligraphy type letter such as V.

## 2. Oblique subspace projections

An oblique subspace projection matrix (ObSP) is a linear operator that projects a given vector onto a target subspace following the direction of a reference subspace. Conversely, an orthogonal subspace projection (OrSP) can be seen as a special case of an ObSP where the target and reference subspaces are orthogonal (Yanai et al., 2011), this permits the construction of OrSP using only a basis for the target subspace. In contrast with ObSP, OrSP is the most popular projection operator since it arises naturally in the least squares solution of a linear regression problem.

In general, in order to construct an oblique projector the basis for the target and the reference subspaces should be known. Let $V\subset {\mathbb{R}}^{N}$ represent the subspace spanned by a matrix **A** ∈ ℝ^*N*×*p*^, where *N* and *p* represents the number of rows and columns of the matrix **A** respectively, $V_{k}\subset V$ the signal subspace spanned by a partition of **A**, **A**_*k*_, with **A** = [**A**_*k*_ **A**_(*k*)_]. If $V=V_{1}⊕V_{2}⊕\ldots ⊕V_{d}$, with ⊕ being the direct sum operator, then the oblique projector onto $V_{k}$ along $V_{\left(k\right)}=V_{1}⊕\ldots ⊕V_{k-1}⊕V_{k+1}\ldots ⊕V_{d}$, denoted by *P*_*k*.(*k*)_, with *d* ≤ *p*, is given by:

(1) $$\begin{array}{l} {\text{P}}_{k.\left(k\right)}={\text{A}}_{k}{\left({\text{A}}_{k}^{T}{\text{Q}}_{\left(k\right)}{\text{A}}_{k}\right)}^{†}{\text{A}}_{k}^{T}{\text{Q}}_{\left(k\right)}, \end{array}$$

where † represents the generalized inverse, *d* represents the number of signal subspaces embedded in **A** and satisfies *d* ≤ *p*, and **Q**_(*k*)_ is the orthogonal projector onto Null$\left({\text{A}}_{\left(k\right)}^{T}\right)\subset V_{\left(k\right)}^{⊥}$, which is computed as:

(2) $$\begin{array}{l} {\text{Q}}_{\left(k\right)}={\text{I}}_{N}-{\text{P}}_{\left(k\right)}, \end{array}$$

where ${\text{P}}_{\left(k\right)}={\text{A}}_{\left(k\right)}{\left({\text{A}}_{\left(k\right)}^{T}{\text{A}}_{\left(k\right)}\right)}^{†}{\text{A}}_{\left(k\right)}^{T}$ is the orthogonal projector onto $V_{\left(k\right)}\equiv$ Span(**A**_(*k*)_) (Yanai et al., 2011).

ObSP arises naturally from the solution of a generalized least squares (GLS) estimation problem. The GLS regression problem is defined as:

(3) $$\begin{array}{cc} \underset{x}{\min} & \epsilon^{T}\Sigma \epsilon \\ \text{s.t. } & \epsilon =y-Ax, \end{array}$$

where **Σ** = *Var*[**ϵ**|**A**], and $\text{A}=\left[{\text{a}}_{1}^{T};\ldots ;{\text{a}}_{N}^{T}\right]$, where **A** ∈ ℝ^*N*×*p*^, with *N* equal to the number of observations. Since the cost function is quadratic in terms of the model parameters **x**, the solution has the following closed form:

(4) $$\begin{array}{l} {\hat{\text{x}}}_{GLS}={\left({\text{A}}^{T}\Sigma^{-1}\text{A}\right)}^{†}{\text{A}}^{T}\Sigma^{-1}\text{y}. \end{array}$$

Substituting Equation (4) in (3) we obtain $\hat{\text{y}}=\text{A}{\left({\text{A}}^{T}\Sigma^{-1}\text{A}\right)}^{†}{\text{A}}^{T}\Sigma^{-1}\text{y}$, which can be written as $\hat{\text{y}}=\text{Z}\text{y}$. The matrix **Z** is idempotent, **Z**^2^ = **Z**, and not symmetric, **Z**^*T*^ ≠ **Z**, which indicates that **Z** is an ObSP matrix, provided $\Sigma^{-1}={\text{Q}}_{\left(k\right)}$ (Yanai et al., 2011).

GLS considers that the noise variance is not constant and mitigates its effects by including a weighting matrix that is obtained from the variance of the estimated noise (Kariya and Kurata, 2004).

The noise has a large influence in the performance of ObSP. Behrens and Scharf (1994), studied the influence of structured and white noise in the projections when using ObSP. They found that, when the signal subspace for the structured noise is known, an ObSP operator can be created such that its kernel contains this subspace, eliminating the influence of the structured noise from the estimation. However, even though ObSP can effectively remove the structured noise, some components from the white noise might be amplified. They concluded that ObSP is more efficient when the structured noise is dominant to the background white noise. Therefore, by reducing the background noise the efficiency of ObSP to remove structured noise can be improved. Normally this would not be a problem when the noise is considered to be another physiological measurement. However, noise can be reflected in small eigenvalues which might inflate the estimation producing results with a high variance. This problem can be mitigated by the use of a regularization term that numerically stabilizes the solution. This approach will be discussed in Section 2.2. First we will discuss the geometrical interpretation for ObSP.

### 2.1. Geometrical interpretation

ObSP operators also appear naturally in the least squares solution of a modified linear regression problem. Consider the linear regression problem **y** = **Ax** + **ϵ**. By multiplying it by **Q**_(*k*)_, defined in Equation (2), we obtain the following regression problem:

(5) $$\begin{array}{l} {\text{Q}}_{\left(k\right)}\text{y}={\text{Q}}_{\left(k\right)}\text{A}\text{x}+{\text{Q}}_{\left(k\right)}\epsilon , \end{array}$$

and its Least Squares solution is then given by:

(6) $$\begin{array}{l} {\hat{\text{x}}}_{LS}={\left({\text{A}}^{T}{\text{Q}}_{\left(k\right)}\text{A}\right)}^{-1}{\text{A}}^{T}{\text{Q}}_{\left(k\right)}\text{y}. \end{array}$$

Replacing Equation (6) in (5), and using the ObSP operator in Equation (1) we obtain:

(7) $$\begin{array}{l} {\text{Q}}_{\left(k\right)}\text{y}={\text{Q}}_{\left(k\right)}{\text{P}}_{k.\left(k\right)}\text{y}+{\text{Q}}_{\left(k\right)}\epsilon . \end{array}$$

**Figure 1:**
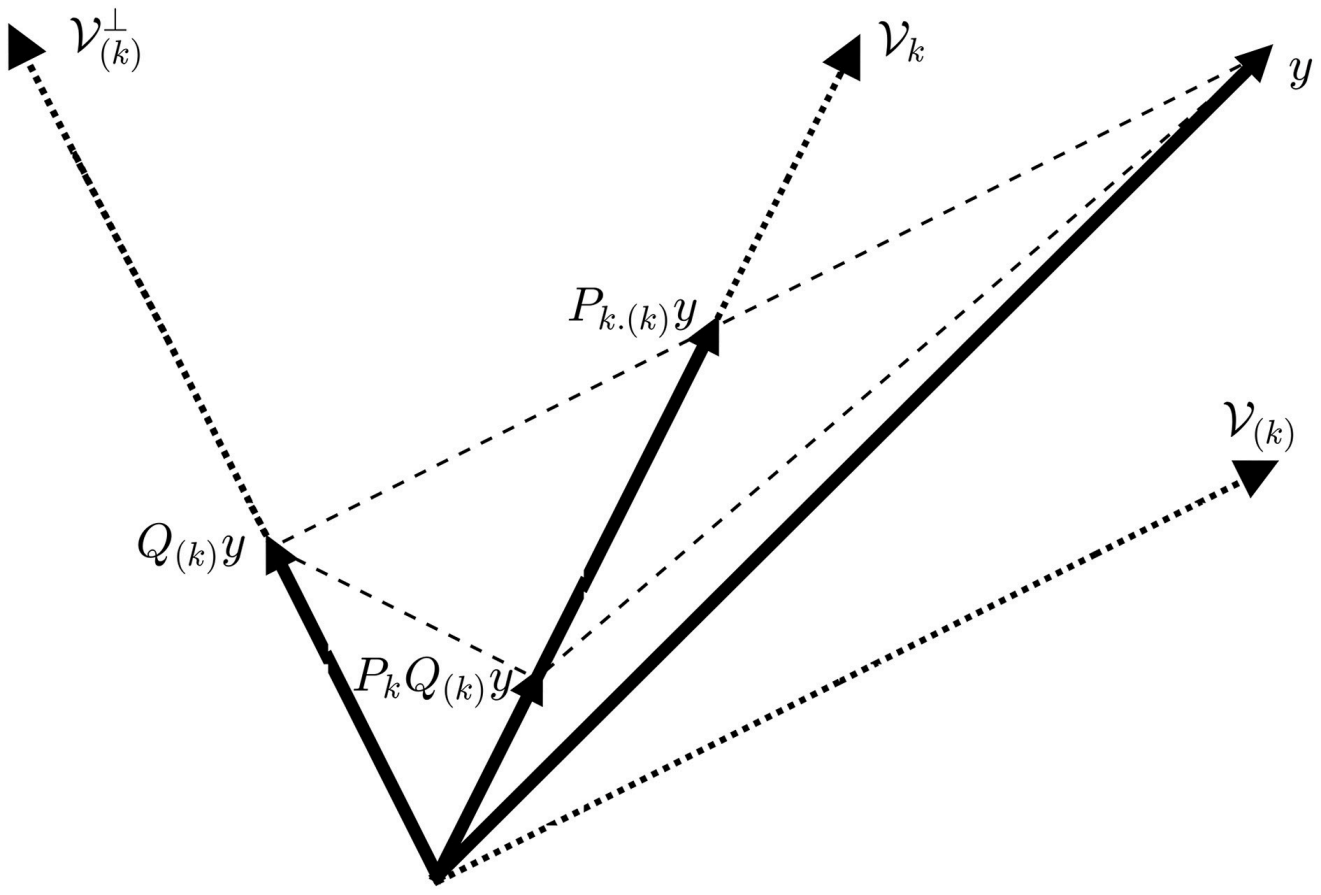
**Geometrical interpretation of the ObSP operator**. The dotted lines represent subspaces, the dashed lines represent the path followed for the different projections, and the solid lines represent vectors.

Based on Equation (7), we can interpret an ObSP operator as a linear mapping of a given vector, **y**, onto a desired subspace, $V_{k}\equiv$ Span{**A**_*k*_}, following a reference subspace, $V_{\left(k\right)}\equiv$ Span{**A**_(*k*)_}, such that the mapped vector, **P**_*k*.(*k*)_**y**, has the same orthogonal projection onto the complement of the reference subspace, $V_{\left(k\right)}^{⊥}\equiv$ Null$\left\{{\text{A}}_{\left(k\right)}^{T}\right\}$, as the vector **y**; notice that **A**_*k*_ ⊄ **A**_(*k*)_. This can be clearly seen in Figure 1.

Interestingly, the least squares solution to the problem in Equation (5), **x**_*LS*_, is the same as the solution to

(8) $$\begin{array}{l} {\text{P}}_{k.\left(k\right)}\text{y}={\text{P}}_{k.\left(k\right)}\text{A}\text{x}+{\text{P}}_{k.\left(k\right)}\epsilon . \end{array}$$

This is proven as follows:

*PROOF*. The Least Squares solution to Equation (5) is equal to ${\hat{\text{x}}}_{LS}={\left({\left({\text{Q}}_{\left(k\right)}\text{A}\right)}^{T}{\text{Q}}_{\left(k\right)}\text{A}\right)}^{-1}{\left({\text{Q}}_{\left(k\right)}\text{A}\right)}^{T}y$, since ${\text{Q}}_{\left(k\right)}^{T}{\text{Q}}_{\left(k\right)}={\text{Q}}_{\left(k\right)}$, **Q**_(*k*)_**A** = **Q**_(*k*)_**A**_*k*_, and ${\text{Q}}_{\left(k\right)}^{T}={\text{Q}}_{\left(k\right)}$ then the solution simplifies to ${\hat{\text{x}}}_{LS}={\left({\text{A}}_{k}^{T}{\text{Q}}_{\left(k\right)}{\text{A}}_{k}\right)}^{-1}{\text{A}}_{k}^{T}{\text{Q}}_{\left(k\right)}y$. Similarly, solving Equation (8), we obtain ${\hat{\text{x}}}_{LS}={\left({\left({\text{P}}_{k.\left(k\right)}\text{A}\right)}^{T}{\text{P}}_{k.\left(k\right)}\text{A}\right)}^{-1}{\left({\text{P}}_{k.\left(k\right)}\text{A}\right)}^{T}{\text{P}}_{k.\left(k\right)}y$. Since **P**_*k*.(*k*)_**A** = **A**_*k*_, and *P*_*k*.(*k*)_ is defined in Equation (1), simplifying the equations we obtain ${\hat{\text{x}}}_{LS}={\left({\text{A}}_{k}^{T}{\text{Q}}_{\left(k\right)}{\text{A}}_{k}\right)}^{-1}{\text{A}}_{k}^{T}{\text{Q}}_{\left(k\right)}y$.

### 2.2. ObSP and tikhonov regularization

Tikhonov regularization is the most general form of the Ridge regularization, where a linear regression problem is formulated as follows (Golub et al., 1999):

(9) $$\begin{array}{l} \begin{array}{cc} \underset{\text{x}}{\min} & \epsilon^{T}\epsilon +\gamma ||\Gamma \text{x}|{|}_{2}^{2}\\ \text{s.t.}\; & \epsilon =\text{y}-\text{A}\text{x}, \end{array} \end{array}$$

when the matrix **Γ** = **I**, the problem is known as Ridge regression. Tikhonov regularization is used in order to impose some kind of property to the solution vector **x**. In this manuscript the solution vector **x**, due to the construction of the matrix **A**, will represent the impulse response of a physiological system. Therefore, we are interested in imposing smoothness to the estimated output of the linear problem, since it is expected for this response to be smooth. In this context, the regularization matrix **Γ** will take the form of a differential operator, with entries such as **Γ**(*i, i*) = −1 and **Γ**(*i, i* + 1) = 1. In order to obtain a regularized output for the oblique projections we can solve the equivalent problem presented in Equation (5):

(10) $$\begin{array}{cc} \underset{x}{\min} & {\hat{\epsilon }}^{T}\hat{\epsilon }+\gamma \|\Gamma x{\|}_{2}^{2}\\ \text{s.t. } & \hat{\epsilon }=Q_{\left(k\right)}y-Q_{\left(k\right)}Ax, \end{array}$$

with ϵ^=Q(k)ϵ. The solution, $\hat{x}={\hat{x}}_{ObSP}$, is given by:

(11) $$\begin{array}{l} {\hat{\text{x}}}_{ObSP}={\left({\text{A}}_{k}^{T}{\text{Q}}_{\left(k\right)}{\text{A}}_{k}+\gamma \Gamma^{T}\Gamma \right)}^{-1}{\text{A}}_{k}^{T}{\text{Q}}_{\left(k\right)}\text{y}, \end{array}$$

where **A**_*k*_ represents a partition of **A** containing only the columns that contains the *k*th regressor, **Q**_(*k*)_ is computed using Equation (2), γ is the regularization constant, and **Γ** represents the regularization matrix. This problem does not require the computation of ObSP projectors, which are more costly than the construction of orthogonal projectors. In addition, since the norm of oblique projectors might be larger than one, resulting in the possible amplification of noisy components, solving Equation (11) is numerically more stable.

## 3. Signal decomposition based on oblique subspace projections (SIDE-ObSP)

Let's consider *N* observations from a linear time-invariant (LTI), multiple-input and single-output (MISO) system, with output **y** and *d* input variables $\text{S}={\left\{{\text{s}}_{i}\right\}}_{i=1}^{d}$, with ${\text{s}}_{i}\in {\mathbb{R}}^{N}$. The output of this system in discrete time can be represented as follows:

(12) $$\begin{array}{l} y\left[n\right]=\overset{d}{\underset{i=1}{\sum }}\overset{p_{i}}{\underset{m=0}{\sum }}{\text{s}}_{i}\left[n-m\right]{\text{h}}_{i}\left[m\right]+\epsilon , \end{array}$$

where **h**_*i*_ represents the impulse response of the subsystem that links the input variable **s**_*i*_ and its corresponding contribution to the output **y**_*i*_, *p*_*i*_ is the length of the impulse response, and ϵ is the background noise. Notice that the number of input variables, *d*, also represents the number of signals subspaces spanned by the system. If the system is stable its impulse response, **h**, decays toward zero. Therefore, **h** can be truncated by an appropriate sample number *p*, such that *h*_*i*_[*n*] > δ ∀*i*, with δ being an appropriately chosen threshold, and *n* = {1, …, *p*}, the selection of *p* will be discussed later. Then, the model in Equation (12) can be represented as a linear regression problem of the form:

(13) $$\begin{array}{l} \text{y}=\text{A}\text{h}+\epsilon , \end{array}$$

where **y** ∈ ℝ^*N*^ is the output of the system, **A** ∈ ℝ^*N*×(*dp*)^ is the regressor matrix that represents the signal subspaces, and **h** ∈ ℝ^*dp*^ is a vector containing the concatenated impulse responses ${\text{h}}^{T}={\left\{{\text{h}}_{i}^{T}\right\}}_{i=1}^{d}$. Using the input matrix **S** = [**s**_1_, …, **s**_*d*_] and approximating **y** as the output of a moving average (MA) system with finite impulse response, we construct the matrix **A** as a block Hankel expansion of the input matrix **S**, using *p* delayed samples from each input variable, **s**_*i*_, in order to define its signal subspace.

Given a vector ${\text{s}}_{k}\in {\mathbb{R}}^{N}$, a “Hankel” matrix constructed from **s**_*k*_ consists of forming a square matrix **A**_*k*_, such that its entries follow **A**_*k*_(*i, j*) = **A**_*k*_(*i*−1, *j* + 1). The columns of the matrix **A**_*k*_ are delayed versions of the vector **s**_*k*_. In the proposed framework, the matrix **A**_*k*_ is a truncated version where the number of columns per each input signal is limited to the order of the MA model, *p*. This matrix is called a block Hankel matrix. The order *p* can be found using cross-validation, or can be set to a sufficiently large number such that all the impulse responses at the sample number *p* are smaller in magnitude than a selected threshold. The matrix **A** can be obtained by concatenaiting the matrices **A**_*k*_ obtained by the block Hankel expansion of the input signals **s**_*k*_ for *k* = {1, …, *d*}, such that **A** = [**A**_1_, …, **A**_*d*_].

Since we are interested in finding the linear contribution of each input variable **s**_*i*_ on the output **y**, we can reformulate the model in Equation (13) as follows:

(14) $$\begin{array}{r} \text{y}={\text{A}}_{k}{\text{h}}_{i}+{\text{A}}_{\left(k\right)}{\text{h}}_{\left(i\right)}+\epsilon , \end{array}$$

where **A**_*k*_ is the partition of **A** that is related to the input **s**_*i*_, and it spans the signal subspace of that input. Notice that due to the Hankel expansion of the matrix **S**, the signal subspace corresponding to the *i*th input variable, **s**_*i*_, now corresponds to the *k*th partition of the matrix **A**, ${\text{A}}_{k}\in {\mathbb{R}}^{N\times p}$. ${\text{h}}_{i}\in {\mathbb{R}}^{p}$ represents the impulse response of the system linked to the input **s**_*i*_, ${\text{A}}_{\left(k\right)}\in {\mathbb{R}}^{N\times \left(d-1\right)p}$ represents the remaining columns of **A**, and ${\text{h}}_{\left(i\right)}\in {\mathbb{R}}^{\left(d-1\right)p}$ is the vector containing the impulse responses of the remaining subsystems. In order to eliminate the influence of the undesired inputs, we can project **y** onto the Span{**A**_*k*_}. However, the subspace spanned by the residual components **A**_(*k*)_ = [**A**_1_, …, **A**_*k*−1_, **A**_*k*+1_, …, **A**_*d*_] is likely to be oblique to the Span{**A**_*k*_}. Notice that **A**_(*k*)_ is obtained by concatenating the block Hankel expansion of all the inputs except the *k*th input. To solve this problem we can modify it by multiplying Equation (14) by **Q**_(*k*)_, computed as in Equation (2). This results in:

(15) $$\begin{array}{l} \begin{array}{ccc} {\text{Q}}_{\left(k\right)}\text{y} & = & {\text{Q}}_{\left(k\right)}\left({\text{A}}_{k}{\text{h}}_{k}+{\text{A}}_{\left(k\right)}{\text{h}}_{\left(k\right)}+\epsilon \right),\\ = & {\text{Q}}_{\left(k\right)}{\text{A}}_{k}{\text{h}}_{k}+\eta_{k}, \end{array} \end{array}$$

where **Q**_(*k*)_**y** can be seen as a preprocessed version of **y**, where the linear contribution of the regressors **A**_(*k*)_ has been eliminated, and **η**_*k*_ = **Q**_(*k*)_**ϵ** is the residual noise component. The solution of this problem using the Tikhonov regularization is given by Equation (11). The linear contribution of the *i*th input regressor can be found as ${\hat{\text{y}}}_{i}={\text{A}}_{k}{\hat{\text{h}}}_{i}$. Since this is equivalent to ${\hat{\text{y}}}_{i}={\text{P}}_{k.\left(k\right)}\text{y}$, and taking into account that oblique projectors might amplify noisy components, as mentioned before, we should guarantee that the structural noise is larger than the background noise to avoid these problems. In the framework of decomposition of biomedical signals, we are interested in removing physiological interferences, hence the structural noise will consist of physiological measurements that normally have a higher power than the background noise.

The decomposition algorithm proposed in this paper, called from now on SIDE-ObSP, is summarized in the Algorithm presented below.

Since the column subspace of the oblique projector *P*_*k*.(*k*)_ is spanned by the dynamics of the *k*th input variable, and its Null subspace is represented by the signal subspace from all the other inputs, the oblique projector is able to decouple the dynamics of the *k*th input from all the other variables, even in the presence of high correlations among them. However, it is important to notice than in the pathological case when the signal subspace of the *k*th variable is also contained in the Null subspace of the remaining inputs, then the problems becomes ill-defined and the projection matrix tends to infinite. In practice this is counter intuitive, because in this case the Column subspace of the projector will be contained in its Null subspace. In the case of extremely high correlated signals, this might lead to numerical problems that affect the solution, due to the presence of the inverse in Equation (6), however, the use of Tikhonov regularization should be able to partially mitigate this problem as can be seen from Equation (11).

**Algorithm:** **SIgnal DEcomposition based on Oblique Subspace Projections (SIDE-ObSP)**.

**Input:** regressor matrix **S** ∈ ℝ^*N*×*d*^, output vector **y** ∈ ℝ^*N*^.	
**Output:** Matrix formed with the decomposition of the output vector $\hat{\text{Y}}=\left[{\hat{\text{y}}}_{1},\ldots ,{\hat{\text{y}}}_{d}\right]$, with $\hat{\text{Y}}\in {\mathbb{R}}^{\left(N-p\right)\times d}$.	
1.	Using the input matrix **S** create the matrix **A**, by using a block Hankel expansion of **S** with an appropriate order *p*. This order can be set at the beginning using prior knowledge or can be set automatically using cross-validation.
2.	For each input variable partition the input matrix as follows **A** = [**A**_*k*_ **A**_(*k*)_], where the partition **A**_*k*_ consists of all the columns related to the *i*th input **s**_*i*_.
3.	Using **A**_(*k*)_ compute **Q**_(*k*)_, as in Equation (2), and postulate the regression problem as shown in Equation (15).
4.	Using cross-validation compute the adequate regularization constant γ and the order of the MA system *p*. When using cross-validation evaluate the cross-validation error as $e=\frac{1}{N_{v}}||{\text{Q}}_{\left(k\right)}\text{y}-{\text{Q}}_{\left(k\right)}{\text{A}}_{k}{\text{h}}_{i}|{|}_{2}^{2}+\frac{1}{p}||\Gamma {\text{h}}_{i}|{|}_{2}^{2}$, with *N*_*v*_ the number of data points used in the validation set. This guarantees smoothness in **h**_i_^*^.
5.	Compute the estimated linear contribution of the *i*th regressor on the output as ${\hat{\text{y}}}_{i}={\text{A}}_{k}{\hat{\text{h}}}_{i}$.
6.	Concatenate all the outputs, ${\hat{\text{y}}}_{i}$, in the matrix $\hat{\text{Y}}=\left[{\hat{\text{y}}}_{1},\ldots ,{\hat{\text{y}}}_{d}\right]$.

For numerical stability *N* ≫ *dp*. A rule of thumb for the number of observations needed is to use 70% of the available data to compute the model parameters and 30% for validation. Taking this into account the number of data points needed for the algorithm, defining an upper limit for *p* = *P*, will be *N* = 10*dP*, and *N*_*v*_ = 4*dP*.

## 4. Applications

### 4.1. Simulation study

We considered *N* = 1024 observations {*y*_*i*_, **x**_*i*_}, with *y*_*i*_ ∈ ℝ and ${\text{x}}_{i}\in {\mathbb{R}}^{3}$, following the model **y** = *f*_1_(**x**_1_) + *f*_2_(**x**_2_) + *f*_3_(**x**_3_) + **η**, where **η** is uniformly distributed random noise with zero mean, and a chosen standard deviation such that we obtain a signal-to-noise ratio *SNR* = 4*db* in the signal **y**. The function *f*_1_ was selected as a 3rd order low-pass Butterworth filter, with normalized frequency of 0.15 *half-cycles*/*sample*, the functions *f*_2_, and *f*_3_ had normalized frequencies of [0.1–0.2] *half-cycles*/*sample* and [0.05–0.15] *half-cycles*/*sample*, respectively.

We considered the inputs {**x**_1_, **x**_2_, **x**_3_}, to be three binary pseudorandom signals (PBRN) in the normalized frequency range [0–0.3] *half-cycles*/*sample*. In addition we induced a correlation of ρ = 0.5 between the input signals by adding a 4th reference PBRN signal, **x**_4_, to all the inputs using the following formula:

(16) $$\begin{array}{l} {\text{x}}_{i}=\rho {\text{x}}_{4}+\left(\sqrt{1-\rho^{2}}\right){\text{x}}_{i};\;i=\left\{1,2,3\right\}. \end{array}$$

Before applying SIDE-ObSP, we also contaminated all the inputs with uniformly distributed random noise *N*(0, σ^2^), where σ was chosen to reach a *SNR* = 4db. In addition, to complicate more the decomposition problem, we filtered the signal **x**_4_ using a low pass Buttherworth filter of 3rd order and normalized cut-off frequency of 0.3 *half-cycles*/*sample*, the filtered signal was mixed together with the output signal **y**, imposing a correlation of 0.3 between them, using Equation (16). We applied ObSP using the noisy inputs, **x**_*i*_ and the noisy output **y** to find the linear contribution of each input on the output, **y**_*i*_ = *f*_*i*_(**x**_*i*_).

**Figure 2:**
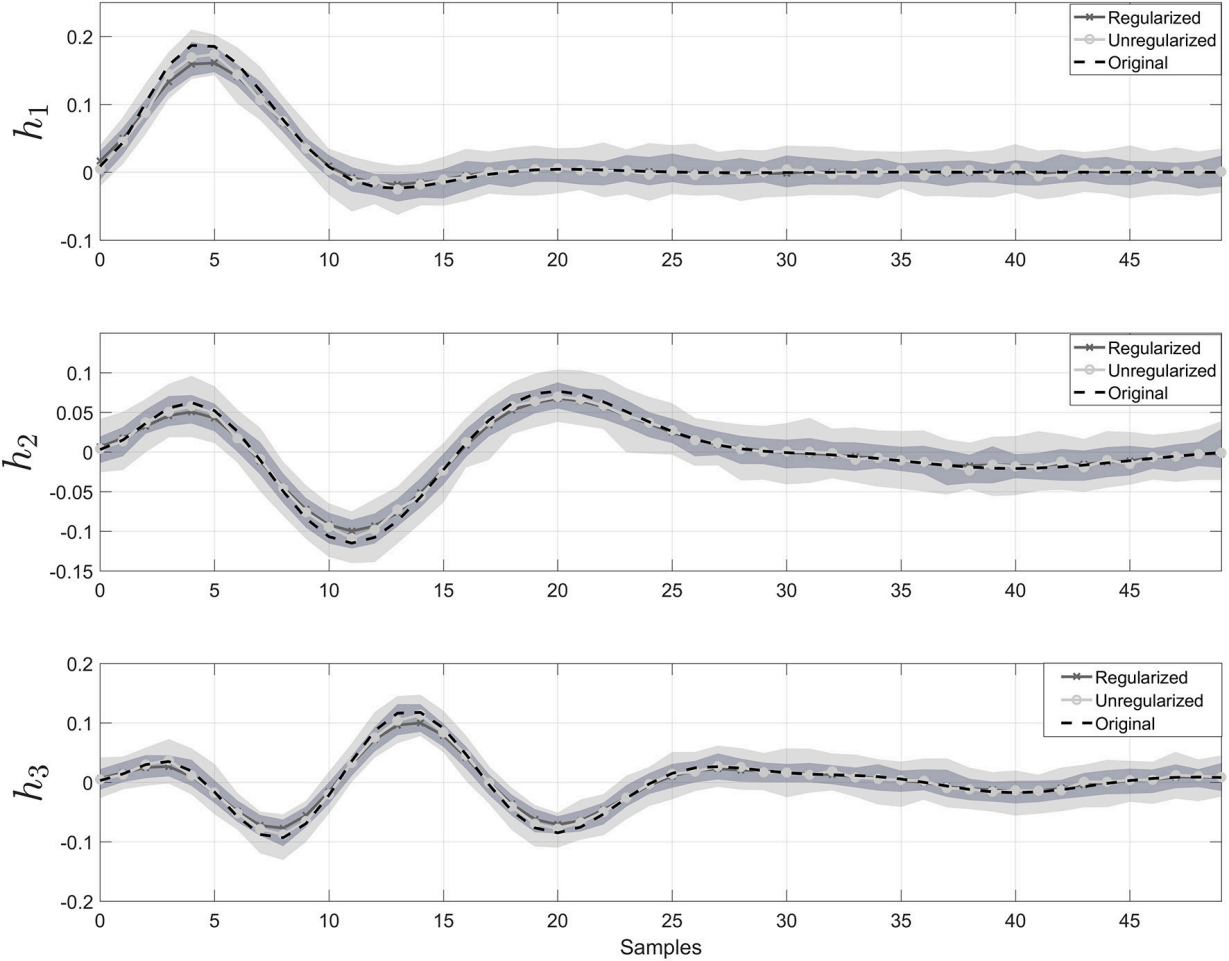
**Impulse response for the three different filters**. The dashed black line represents the reference impulse response. The solid gray line with “x” symbols represents the output of SIDE-ObSP using regularization, and the solid lighter line with filled circles represents the output without regularization. The light gray shadow represents the 95% confidence intervals for SIDE SIDE-ObSP without regularization, while the darker gray shadow area represents the 95% confidence interval for the regularized version of SIDE-ObSP. Regularized SIDE-ObSP produces smoother estimates with smaller confidence intervals. The order of the MA model, for all the 100 repetitions was fixed to *m* = 50, and the regularization constant γ, was found using 10-fold cross-validation.

In Figure 2, the estimated impulse responses of the sub-systems, $\hat{{\text{h}}_{i}}$, using SIDE-ObSP and its unregularized version are shown. We performed 100 simulations and plotted the median and the 95% confidence interval for the estimated impulses responses using regularized and unregularized SIDE-ObSP. It can be seen that regularization smooths the estimations of the impulse response of the systems.

We compared the output from SIDE-ObSP with the output of four different system identification model structures: subspace system identification, ARMAX, ARX and an adaptive filtering model. We used the *System Identification* toolbox from MATLAB in order to estimate the model. For the subspace system identification we used the function “*n4sid”* in MATLAB, using zero delays in the input variables and evaluating the order of the system between 2 and 8, since the order of the systems to be identified lie within this range, the final order of the model was selected based on the suggestion given by the algorithm output. For the ARX model, we evaluated different model orders and selected the order that minimizes the Akaike information criteria, as proposed by the function *selstruc*. For the ARMAX model we fixed the model parameters to [*n*_*a*_, *n*_*b*_, *n*_*c*_, *n*_*k*_] = [5, 5, 5, 0]^1^. We checked that the selected order for the ARMAX models produce a satisfactory output. Finally, for the adaptive filtering approach, we used the function *adaptfilt* from MATLAB, using the *LMS FIR adaptive filter algorithm*. The length of the adaptive filter was selected as the identified optimal order of SIDE-ObSP; in addition, the LMS step size was set to 0.008.

To evaluate if SIDE-ObSP is able to effectively decompose the output measurement in a set of linear contributions from each input variable, and if it outperforms the tested system identification models, we computed the estimated output for each one of the three different sub-systems, using the different methods, and computed the cross-entropy between the estimated linear contributions on the output from each input and the different inputs. In short, cross-entropy is a measure of the information transfer between 2 signals. A cross-entropy value equal to 0 indicates no information transfer, cross-entropy values different from zero indicate the amount of information that is transferred from one signal to another, with larger values indicating stronger transfer. For more details on this measure we refer the reader to Faes et al. (2011). We will use from here on the information transfer as a measure for the coupling between the variables. The results are presented in Table 1. It can be seen that SIDE-ObSP increases the coupling between each input variable and their corresponding estimated linear influence, whilst reducing the coupling with other input variables. The bold numbers indicate the values of cross-entropy which are different from zeros, i.e., they indicate the pair of signals which present at some degree a linked dynamics. As can be seen in the table only the diagonal elements, in the case of the output provided by SIDE-ObSP, present cross-entropy values different from 0. The other algorithms fail to decouple the linear contributions, since cross-entropy values different from zero can be seen in the off-diagonal elements for each one of the other methods, indicating the presence of some degree of coupling between input variable and the different estimated linear contributions. In addition, the larger cross-entropy values indicate stronger coupling between the variables. Only the outputs from the subspace system identification model produce larger cross-entropy values between the input signals and their respective contributions when compared to the output from SIDE-ObSP. This might be attributed to the fact that the solutions provided by subspace system identification are less noisy. However, subspace system identification is not able to completely decouple the influence of other inputs onto each partial linear contribution.

**Table 1:** **Cross-entropy values between each input variable, x_*i*_, and the estimated linear contributions, ${\hat{\text{y}}}_{i}$, for ARX, ARMAX, adaptive filtering (Adap.), and subspace System identification (SS) models and SIDE-ObSP**.

	**${\hat{\text{y}}}_{\text{1}}^{\text{S}IDE-ObS\text{P}}$**	**${\hat{\text{y}}}_{\text{2}}^{\text{S}IDE-ObS\text{P}}$**	**${\hat{\text{y}}}_{\text{3}}^{\text{S}IDE-ObS\text{P}}$**	**${\hat{\text{y}}}_{\text{1}}^{\text{A}R\text{X}}$**	**${\hat{\text{y}}}_{\text{2}}^{\text{A}R\text{X}}$**	**${\hat{\text{y}}}_{\text{3}}^{\text{A}R\text{X}}$**	**${\hat{\text{y}}}_{\text{1}}^{\text{A}RMA\text{X}}$**	**${\hat{\text{y}}}_{\text{2}}^{\text{A}RMA\text{X}}$**	**${\hat{\text{y}}}_{\text{3}}^{\text{A}RMA\text{X}}$**
**x**_**1**_	**1.0775**	0.0000	0.0000	**0.3716**	**0.0129**	0.0016	**0.4154**	**0.0153**	0.0027
**x**_**2**_	0.0000	**0.5962**	0.0000	**0.0148**	**0.3319**	**0.0158**	**0.0119**	**0.3954**	**0.0116**
**x**_**3**_	0.0000	0.0001	**0.8064**	0.0010	**0.0152**	**0.4495**	0.0008	**0.0168**	**0.3961**
	${\hat{\text{y}}}_{1}^{Adap.}$	${\hat{\text{y}}}_{2}^{Adap.}$	${\hat{\text{y}}}_{3}^{Adap.}$	${\hat{\text{y}}}_{\text{1}}^{\text{S}\text{S}}$	${\hat{\text{y}}}_{\text{2}}^{\text{S}\text{S}}$	${\hat{\text{y}}}_{\text{3}}^{\text{S}\text{S}}$			
**x**_**1**_	0.0004	0.0025	**0.0045**	**2.2313**	0.0029	**0.0097**			
**x**_**2**_	**0.0060**	0.0007	0.0035	**0.0093**	**1.4360**	0.0034			
**x**_**3**_	0.0019	0.0009	0.0020	**0.0075**	**0.0122**	**1.8651**			

The bold numbers indicate values of cross-entropy which are statistically different from zero.

### 4.2. Removal of physiological artifact from NIRS signals

In this section we use SIDE-ObSP to decouple the influence of the variations in SaO_2_ from the tissue oxygenation index (TOI), facilitating the use of TOI as a surrogate measurement of CBF for the assessment of cerebral Autoregulation (CA). TOI represents the ratio between oxygenated hemoglobin and total hemoglobin in the tissue. TOI is measured using spatially resolved spectroscopy as indicated in Suzuki et al. (1999). CA is a mechanism that tries to maintain a more or less stable CBF, despite the changes in MABP (Lassen, 1959). However, it has been shown that CA is not as simple as initially thought, and it comprises a set of responses from different mechanisms related to myogenic, neurogenic and metabolic actions (Panerai, 1998). Monitoring CA is important in order to avoid brain damage due to ischemia and/or hemorrhage (Wong et al., 2008; Soul et al., 2007). TOI can be used as a non-invasive monitoring variable for CBF, it allows the continuous bedside monitoring of CA (Caicedo et al., 2011). However, TOI only reflects changes in CBF under a constant brain metabolism and constant arterial oxygen saturation (SaO_2_). In premature infants, even thought it is a strong statement, the first assumption can be considered valid during the first 3 days of life in the periods of analysis, which normally involve segments of 15 min. But, for the second assumption, the premature infants are likely to suffer from variations in systemic variables, especially in SaO_2_, during their stay at the neonatal intensive care unit (NICU). Under these conditions TOI cannot be used as a robust surrogate for CBF, limiting the assessment of CA using NIRS in clinical practice.

**Figure 3:**
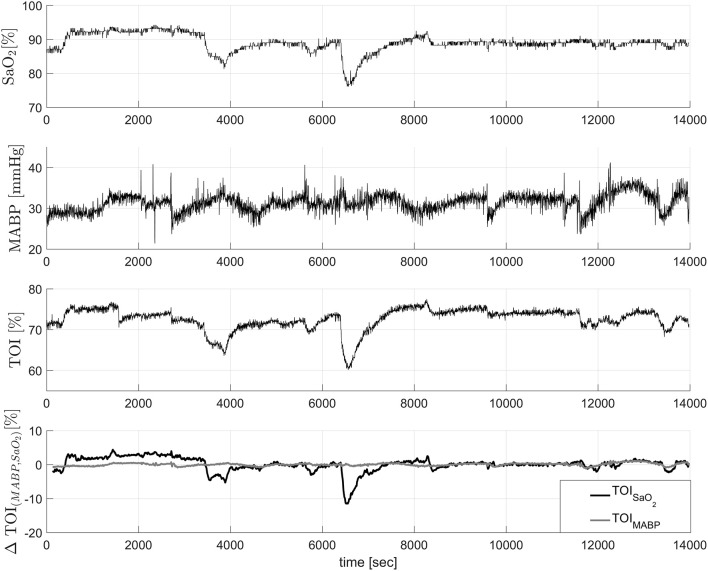
**In the first three figures concomitant measurements of SaO_2_, TOI, and MABP**. It can be clearly seen that the changes in SaO_2_ are reflected in the changes in TOI values. The last figure shows the decomposition of the TOI in components related to changes in SaO_2_ and MABP. The component ΔTOI_MABP_ is not contaminated with the sudden drop caused by the change in SaO_2_. These data was collected from a newborn.

**Figure 4:**
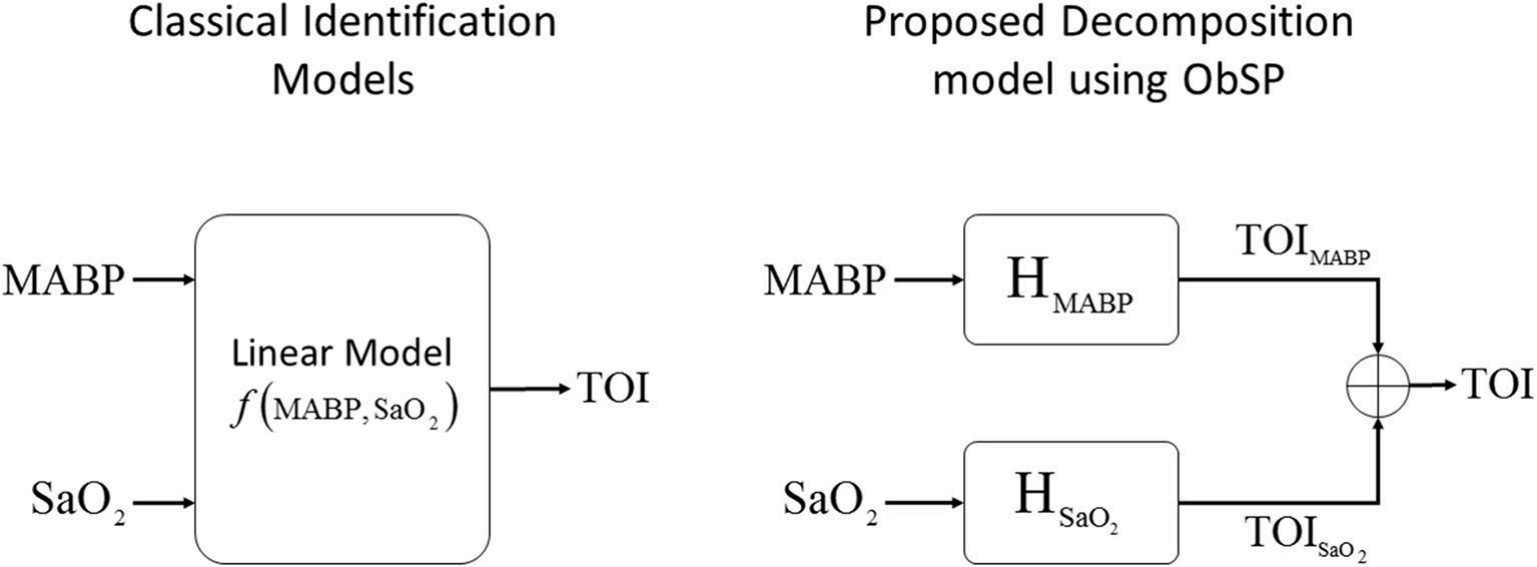
**Schematic representation of the proposed decomposition model, using ObSP, for the removal of physiological influence of SaO_2_ on TOI**. In the left the problem formulation from a classical identification model, in the right the proposed decomposition algorithm SIDE-ObSP.

In this example we used data from 20 infants from the University Hospital Leuven (Belgium), with a gestational age of 28.4±3.5 weeks and a birth weight of 1113 ± 499 g. Neonates were included following approval of the study protocol by the ethical board of the University Hospital, Leuven, Belgium, and after informed written consent was obtained from the parents. In all infants concomitant measurements of SaO_2_, measured by pulse oxymeter (Pulse oxymeter, Novametrix, USA), MABP, measured by an indwelling arterial catheter, and TOI, measured using spatially resolved NIRS (NIRO300, Hamamatsu,Japan), were obtained during the first 3 days of life with a sampling frequency of 1/3Hz. The total length of the recordings is between 6 and 9 h. The measurements were obtained during normal clinical care. For the signal decomposition algorithm, we used the measurements of SaO_2_ and MABP as input variables, and the TOI as output variable. A selected segment of the recordings, where the influence of SaO_2_ is clearly seen, is displayed in the first three panels in Figure 3. The figure shows a sudden drop in SaO_2_ that is reflected in the TOI. We expect that SIDE-ObSP will produce as output the decomposition of TOI into 2 components, such that $\hat{\text{TOI}}={\text{TOI}}_{\text{MABP}}+{\text{TOI}}_{{\text{SaO}}_{\text{2}}}$, where TOI_SaO_2__ is the component related to changes in SaO_2_, and TOI_MABP_ is related to the changes in MABP, as shown in Figure 4.

The results from a representative segment are shown in the last panel of Figure 3. In order to evaluate that TOI_MABP_ and TOI_SaO_2__ are decoupled we used cross-entropy as explained in the previous section. The cross-entropy values between MABP, SaO_2_, TOI, TOI_MABP_ and TOI_SaO_2__ for the representative subject are summarized in Table 2. As in the previous example, these values indicate that TOI_MABP_ and TOI_SaO_2__ are linked mostly to the dynamics of MABP and SaO_2_, respectively, and that there is not cross linked dynamics in the decomposed signals. According to these results we can consider that TOI_MABP_ has been effectively decoupled from the variations in SaO_2_. In addition, based on the cross-entropy values, we can see that the TOI_SaO_2__ has a stronger link to the changes in SaO_2_, than TOI_MABP_ to the changes in MABP. This might indicate that the dynamic in SaO_2_ affects more strongly the changes in TOI than the dynamic of MABP.

**Table 2:** **Cross-entropy values between the input variables and the output generated by SIDE-ObSP**.

	**TOI**	**TOI_MABP_**	**TOI_SaO_2__**
MABP	0.0013	**0.99**	0.0001
SaO_2_	**0.4224**	0.0006	**1.27**

The bold numbers indicate values of cross-entropy which are statistically different from zero.

The results from the complete population are summarized in Table 3. It can be seen that cross-entropy values between SaO_2_/MABP and TOI_SaO_2__/TOI_MABP_ are larger than the cross-entropy values between SaO_2_/MABP and TOI_MABP_/TOI_SaO_2__. This indicate that the influence of other input variables in a given partial contribution are minimized. It is important to note that some of the cross-entropy values between an input and its respective partial contribution are low. Possible explanations for this can be that segments with a low variability in SaO_2_ and MABP are not able to produce a proper description of the signals subspaces; that the segment under analysis is not coupled to the dynamics of the input signals, therefore the model fails to produce a component that is linearly related to the given input; or that the causal relationship, input vs. output, imposed in the model does not hold.

**Table 3:** **Cross-entropy values between the input variables and the output generated by SIDE-ObSP for the complete studied population**.

	**TOI**	**TOI_MABP_**	**TOI_SaO_2__**
MABP	0.1211	0.5853	0.0003
	[0.0001–1.3676]	[0.0170–3.0900]	[0.0000–0.1001]
SaO_2_	0.0261	0.0009	0.6351
	[0.0002–0.6028]	[0.0000–0.1733]	[0.0453–2.1361]

Values given as median[min-max].

In the context of cerebral autoregulation assessment, TOI_MABP_ can be used, directly, to assess the status of the CA mechanism. This component not only represents a version of TOI decoupled from the variations in SaO_2_, but also represents the component of TOI that is linearly linked to the variations in MABP. SIDE-ObSP clearly offers the possibility of using NIRS for the assessment of cerebral autoregulation, even in the presence of changes in SaO_2_. This is an important result with a potentially high clinical impact that needs to be evaluated in further studies.

### 4.3. Brain hemodynamics monitoring

In this section we use SIDE-ObSP in order to decompose the changes in cerebral and peripheral oxygenation into the partial contributions of each systemic variable, in order to evaluate the physiological responses caused by them, independently, in the peripheral and the cerebral circulation. We use a set of measurements obtained from a 3 years old infant under veno-arterial Extra corporeal Membrane Oxygenation (ECMO) procedure. ECMO is used to provide cardio-respiratory support to children with cardiac and/or respiratory problems. During this procedure the main vessels in the neck (right internal jugular vein and carotid artery) are cannulated. Blood is passed to an external oxygenator (via the vein cannula) and pumped back to the heart (via the arterial cannula). The heart and lungs are bypassed and allowed time to rest until they recover. Once there is indication that the patient's heart and lungs have recovered, they are being weaned off ECMO. During weaning, the ECMO blood flow is sequentially reduced, allowing the patients heart and lungs to take over. If during the weaning phase the patients can sustain normal function of their own heart and lungs they are decannulated and completely removed from ECMO. Patients under ECMO are at high risk of hemodynamic instability due to the possible alteration of the regulation mechanisms caused by multi-factorial reasons such as heparinitazion, hemodilution, and reduced arterial pulsatility (Annich et al., 2012).

These data was collected for research purposes, and the collection of the data was approved by the UCL, Institute of Child Health and Great Ormond Street Hospital for Children NHS Trust Research Ethics Committee. Written informed parental consent was obtained from the participants prior to inclusion. Cerebral tissue oxygenation (cTOI) was measured using a NIRS system (NIRO 200, Hamamatsu Photonics KK), using an optode located in the child's forehead; peripheral tissue oxygenation (pTOI) was measured in the calf using the same NIRS system. Concomitant measurements of MABP, end tidal CO_2_ (EtCO_2_), Heart Rate (HR), SaO_2_, and ECMO flow were also recorded during the ECMO weaning phase. Measurements were done during stepwise changes in the ECMO flow; the flow was reduced from baseline (100% ECMO flow) in steps of 10%, approximately every 10 min, until 70% of the baseline ECMO flow was reached, afterward the flow was increased back to baseline following the same profile (Caicedo et al., 2013b).

**Figure 5:**
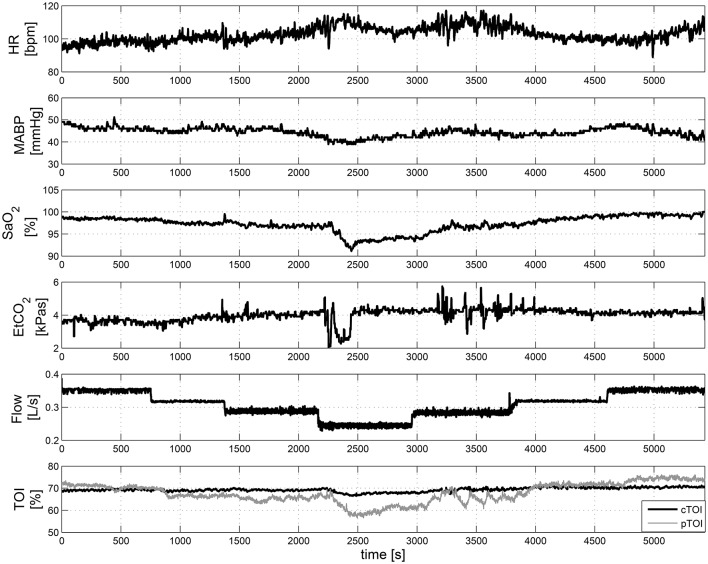
**Measurements of systemic and hemodynamic variables during ECMO “weaning.”** From top to bottom HR, MABP, SaO_2_, EtCO_2_, ECMO Flow, and cTOI, pTOI. These data was collected in a 3-years old infant.

Figure 5 displays some of the raw measurements taken during ECMO weaning. The upper plots in Figure 5 shows the changes in HR, MABP, SaO_2_, EtCO_2_, and the ECMO Flow. The last plot presents the variations in cTOI and the pTOI.

**Figure 6:**
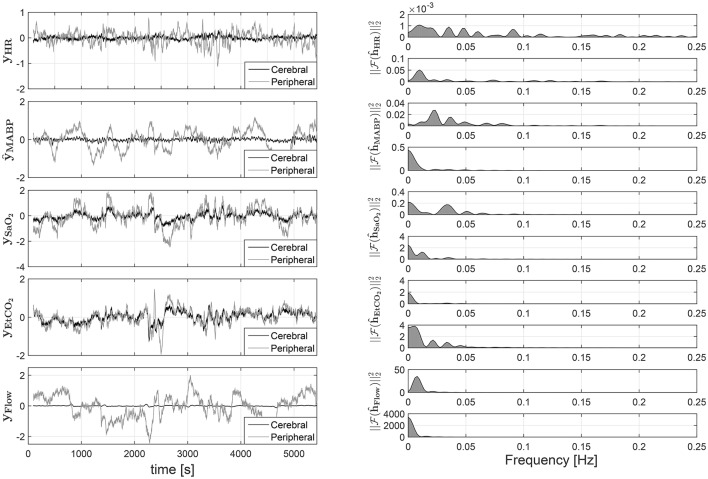
**On the left partial linear contributions of the systemic variables on the cTOI, gray solid line, and pTOI, black solid line, estimated using SIDE-ObSP**. From top to bottom, partial contribution from HR, MABP, SaO_2_, EtCO_2_, and ECMO flow. On the right the frequency response of the individual subsystems linking each input with the output. This response was computed as the amplitude of the Fourier transform of the estimated impulse responses obtained from SIDE-ObSP. The frequency responses are organized presenting the one related to the cerebral hemodynamics first followed by the one related to the peripheral hemodynamics, for each input variable.

The results are presented in Figure 6. In the left panel of the figure, the partial contributions of each systemic variable on the cerebral and peripheral circulation are shown. The right panel presents the frequency response for the different subsytems. By identifying the gain and the frequency band for each frequency response, the characteristics of the different mechanisms involved in the regulation of cerebral and peripheral hemodynamics can be compared. For instance, the magnitude of the frequency response can be used in order to estimate how much the changes on one of the systemic variables affect the corresponding hemodynamic variable. A larger magnitude in the frequency response, i.e., larger gain, indicates that the respective hemodynamic variables are more strongly affected by the specific systemic variation. This is also reflected in a partial contribution with a larger amplitude in the time domain. On the other hand, the bandwidth of the system can be computed by identifying the frequency region with a magnitude larger than 0, this information is useful in order to determine the dynamics of the partial contributions in time domain. Taking this information into account the results presented in Figure 6 indicate that, in contrast with the peripheral circulation, the changes in cerebral hemodynamics caused by variations in MABP, HR, and ECMO flow are highly attenuated since the gain values are much smaller in the frequency response that correspond to the cerebral hemodynamics than the one from the peripheral hemodynamics. This is expected, since the regulation mechanisms that preserve brain hemodynamics are stronger than the regulation mechanisms in the peripheral hemodynamics. On the other hand, changes in SaO_2_, and EtCO_2_ seem to affect cerebral and peripheral hemodynamics strongly, even though once again their impact on cerebral hemodynamics is more attenuated. This might indicate, to some degree, a similar regulatory action for changes in blood gases concentrations on both, the peripheral and the cerebral, vascular beds.

## 5. Discussion

We have proposed a decomposition algorithm based on the use of ObSP, SIDE-ObSP, for the representation of NIRS signals into the partial linear contributions of different physiological signals. SIDE-ObSP assumes that the target signal can be modeled as the sum of the output of a set of mean average filters, one per each input variable. By approximating the structure of the model using MA filters, instead of an ARMAX structure, SIDE-ObSP is able to correctly define signal subspaces per each input variable, without contaminating them with the dynamics of other inputs. The decomposition is then achieved by properly defining oblique projectors that project the output signal onto the subspace spanned by a specific input variable. This oblique projector guarantees that the common dynamics between input variables is decoupled. Thereby, separating the partial linear contributions of each input, even if they are correlated.

SIDE-ObSP makes use of Tikhonov regularization using the derivative operator, in its matrix form, in order to produce smoother responses. The selection of the derivative operator was shown to be appropriate for the applications presented. However, other kernels can be easily integrated into the proposed algorithm, such as the tune-correlated (TC) kernel (Chen et al., 2011).

We demonstrated the performance of SIDE-ObSP with a synthetic example as well as in 2 medical applications in the field of cerebral hemodynamic monitoring. In the synthetic dataset we were able to retrieve the underlying impulse responses of the subsystems that generated the data, we compared the results obtained from SIDE-ObSP with other available methodologies and we showed that for this case, SIDE-ObSP outperformed them. In contracts with ARX, ARMAX, filter adaptive models, and subspace system identification, SIDE-ObSP was able to effectively decouple the dynamics between the input variables and the output, allowing to identify more accurately the underlying dynamics of the different subsystems. This is mainly due to the fact that the signal subspace of undesired input variables is contained in the Null subspace of the ObSP projector. In the first application example we use SIDE-ObSP in order to remove the influence of changes in SaO_2_ from the changes in brain oxygenation measured using NIRS. This is a very important application since it facilitates the monitoring of cerebral autoregulation. In the second application example, we were able to provide a quantitative analysis of the differences between the influence of the systemic variables on the peripheral and cerebral hemodynamics thanks to the use of SIDE-ObSP.

Taking advantage of this decomposition scheme, we present the performance of SIDE-ObSP in 2 medical applications related to cerebral hemodynamics monitoring. First, we use SIDE-ObSP in order to filter out the physiological noise introduced by the variations of SaO_2_ on the TOI. This is important for the non-invasive monitoring of cerebral autoregulation (Liem et al., 1995; Soul et al., 2007; Wong et al., 2008). We found that SIDE-ObSP was not only able to decouple the influence of the undesired fluctuations in SaO_2_, but it was also able to project the changes in TOI on the signal subspace spanned by MABP. This projection carries information about the linked dynamics between TOI and MABP, which can be directly used to quantify the status of the cerebral autoregulation mechanism. In addition, in the second application example, we showed that SIDE-ObSP decomposes the cTOI in the partial contributions of several systemic variables. We compared these results with the decomposition of pTOI and it was found, as expected, that the cerebral hemodynamics regulation mechanisms are able to mitigate and react appropriately to the changes in systemic variables in order to keep the brain hemodynamic homeostasis. The main advantage that SIDE-ObSP presents in this application field, is that it facilitates the individual interpretation and analysis of the influences of the changes in different systemic variables on the peripheral and cerebral hemodynamics. Such influences can be used for the analysis of the different mechanisms that are involved in the regulation of cerebral and peripheral hemodynamics. For instance, the frequency response of the sub-mechanism relating the changes in HR and pTOI acts like a band pass filter with a frequency response between 0 and 0.15 Hz, while the sub-mechanism relating changes in HR and cTOI acts as a low pass filter with a cut-off frequency around 0.1 Hz. This indicates that the partial contributions of HR on the cTOI in the time domain are smoother than the ones in the pTOI. However, response to changes in HR are 50 time stronger in the pTOI than in the cTOI, as indicated by the magnitude of the gain in the filters' pass band. Due to their frequency band we hypothesize this mechanism represents the sympathetic influence on the vascular tone of the cerebral capilar bed and the peripheral circulation. In a previous work we have studied the influence of the HR on the cerebral hemodynamics (Caicedo et al., 2014). Due to the assumption of a linear relationship between the HR and the cTOI and pTOI, no influence of the respiration and/or the cardiorespiratory coupling on the pTOI/cTOI has been included in this analysis, since the frequency range of the NIRS signals is restricted to a very low frequency range, we do not expect a large lineal influence from these variables. When comparing the frequency response of the subsystem linking MABP and TOI, it can be observed that the this subsystem behaves like a band-pass filter in the brain, whilst it exhibits a low-pass frequency response in the peripheral hemodynamics. Moreover, the response to changes in MABP are 20 times larger in the leg than in the brain. This indicates that changes in MABP are passively follow in the peripheral circulation, while only a small transient should be observed in the cerebral hemodynamic as response to changes in MABP, this transient behavior is typical from band pass systems, and it is caused by the fact that the DC values are highly attenuated, therefore any perturbation presented in the input should converge to zero. In addition, the frequency range of the frequency response in this subsystem indicates that the pTOI follows passively oscillations with a frequency smaller than 0.01 Hz (period larger than 100 s), while the frequency range that affects cTOI is restricted to frequencies between 0.01 and 0.1 Hz, representing oscillations between 10 and 100 s. Concerning the influence of SaO_2_, even though it affects both the cTOI and the pTOI, it can be seen that its influence is larger on the peripheral hemodynamics, represented by a gain 10 times larger in the frequency response. This response can be attributed to the larger compliance of the cerebral vascular bed, which is able to mitigate larger changes in CBF, and partially mitigate the effect of large changes in SaO_2_ on the cTOI. The partial contributions of EtCO_2_ to cTOI and pTOI are also similar; however, it can be seen that, in the brain, the changes in EtCO_2_ are smoothed out stronger than in the peripheral circulation, this is also reflected in the lower cut-off frequency exhibit in the frequency response, around 0.01 Hz compared to 0.05 Hz respectively. This smoother behavior, like in the case of SaO_2_, can be caused by a higher capillary compliance in the brain than in the periphery. Finally, it is interesting to observe that the brain acts as a band-pass filter in the presence to changes in the ECMO flow, while the peripheral circulation exhibits a low-pass filter behavior. The protection mechanisms in the brain react to sudden changes in the ECMO flow which afterward are regulated, preserving a stable brain hemodynamics, whilst in the periphery these changes are reflected stronger and passively, due to the low-pass filter characteristic. In addition, the response to changes in ECMO flow in the pTOI are 80 time larger than the response in cTOI. These comparison and analysis was possible thanks to the use of SIDE-ObSP. Monitoring of the cerebral hemodynamics regulations mechanisms is critical for the prevention of brain damage and its consequent sequela, however, mathematical tools to properly monitor them are scarce, mainly, due to the complex linked dynamics of all the different mechanisms that are involved in cerebral hemodynamics regulation (Peng et al., 2008). In this context SIDE-ObSP represents one mathematical tool that can be effectively used in this field, in particular SIDE-ObSP can be useful in the study of the hemodynamic low frequency oscillations (LFO), these oscillations have been observed in since more than 150 years ago, but their origin and physiological explanation is still elusive. The complexity for the interpretation of the LFO is attributed to the coupling of different physiological processes (Sassaroli et al., 2012), it is here where SIDE-ObSP can be used in order to identify which systemic variations are more likely to be related to these oscillation, helping to unrevealed their possible origin and their potential link with cerebral autoregulation.

In addition, other algorithms can be used in order to decompose NIRS measurements in sources that might relate to physiological processes. In this context, Santosa et al. (2013) have developed an algorithm to remove noise sources, physiological or external, from functional NIRS (fNIRS) measurements using independent component analysis (ICA). ICA generally requires a set of measurements that are contaminated by common sources, fNIRS fits naturally within this framework, however, for single channel NIRS measurements this condition is not met. In such cases, single channel ICA can be used Davies and James (2007). However, this approach assumes that the sources are sufficiently disjoint in the frequency domain, which is a condition that cannot be imposed in physiological processes. Also the sources that are obtained using ICA might or might not be related to physiological mechanism. In comparison SIDE-ObSP is able to decompose single channels NIRS measurements and relate them to specific physiological measurements.

However, SIDE-ObSP presents some limitations. First of all, it assumes a linear relationship between the input variables and the output, which is likely not to be the case in biomedical systems. But, if the linear component has a strong contribution SIDE-ObSP can still produce relevant results, as proven with the application examples illustrated in the manuscript. Second, SIDE-ObSP is highly dependent on the available amount of information. This is due to the fact that the projectors for a given input variable are computed using information about the signal subspaces spanned by the other input variables. In case that one of these variables is not included, its subspace will be considered orthogonal to the input signal subspace, even if it is not. This might lead to unexplained dynamics on the decomposition residuals. In the context of clinical monitoring this might not be an issue, due to the availability of different biomedical measurements at the bed side. In addition, since the results are interpreted based on the input data available, residuals linked to other physiological sources can be carefully taken into account for further analysis and interpretation. Furthermore, by studying the dynamics of these residuals, other physiological variables that are linked to the dynamics of the output signal can be found. Finally, SIDE-ObSP assumes stationarity in the processed data segment. In the presence of nonstationarities, the computed model will produce a kind of “average” response. This problem can be mitigated by selecting data segments that are short enough to be considered stationary. However, the length of the processed segments should be large enough in order to guarantee that the number of rows in the regressor matrix **A** is larger that the number of columns. Approaches like subspace tracking methods can be used in order to process continuous stationary segments. This adaptation is useful for online monitoring systems, where nonstationarities are likely to occur but the changes in the system response between two consecutive segments are smooth.

## 6. Conclusion

We have presented a new algorithm, SIDE-ObSP, for the decomposition of NIRS signals into the linear partial contributions of a given set of input variables. SIDE-ObSP uses ObSP in order to effectively decoupled the dynamics from different signals. The application examples highlight its potential use in clinical practice, showing that it might help to enhance diagnosis, understanding of underlying (patho-)physiology, as well as to assess treatment. The main advantage of SIDE-ObSP is that it allows the individual interpretation of the relation between each input variable used in the model and the desired output variable. However, it is important to take into account that its performance relies on the quality and availability of the data used to train the model. Results should be interpreted with caution, taking into account the origin of the available measurements that have been used, and if these measurements are a direct reflection of the physiological variables of interest, or if they are surrogate variables that might include dynamics of different physiological systems. SIDE-ObSP can be extended to be used in other fields not exclusively for biomedical applications. SIDE-ObSP can be easily adapted for online monitoring by means of subspace tracking algorithms. Furthermore, due to its formulation, it can easily be adapted to a nonlinear regression framework based on kernels, such as LS-SVM regression models (Suykens et al., 2003).

## Author contributions

AC was involved in the conception and development of the algorithm presented in this manuscript. CV and BH provided intellectual support in the development of the methodology. MP and IT provided the data for the ECMO application study as well as intellectual support in the interpretation of the results. SV was in charge of the overall coordination of this study. All authors participated in the discussion of the results and the preparation, edition, and revision of the manuscript.

### Conflict of interest statement

The authors declare that the research was conducted in the absence of any commercial or financial relationships that could be construed as a potential conflict of interest.

## Footnotes

^1^For the interpretation of the variables [*n*_*a*_, *n*_*b*_, *n*_*c*_, *n*_*k*_], please refer to the documentation of the function *armax* in MATLAB.
